# Comprehensive analysis of DNA methylation for periodontitis

**DOI:** 10.1186/s40729-022-00420-8

**Published:** 2022-05-02

**Authors:** Zengbo Zhao, Huimin Wang, Xiaona Li, Jingya Hou, Yuntian Yang, Hexiang Li

**Affiliations:** Hebei Key Laboratory of Stomatology, Hebei Clinical Research Center for Oral Diseases, School and Hospital of Stomatology, Hebei Medical University, Shijiazhuang, 050017 People’s Republic of China

**Keywords:** DNA methylation, Periodontitis, Gene expression, Bioinformatitics

## Abstract

**Background:**

Periodontitis is an infectious disease, and a risk factor for peri-implantitis that could result in the implant loss. DNA methylation has an essential role in the etiology and pathogenesis of inflammatory disease. However, there is lack of study on methylation status of genes in periodontitis. This study sought to explore the gene methylation profiling microarray in periodontitis.

**Methods:**

Through searching in the Gene Expression Omnibus database, a gene methylation profiling data set GSE173081 was identified, which included 12 periodontitis samples and 12 normal samples, respectively. Thereafter, the data of GSE173081 was downloaded and analyzed to determined differentially methylated genes (DMGs), which then were used to perform Gene Ontology analysis and pathway enrichment analyses through online database. In addition, the DMGs were applied to construct the protein–protein interaction (PPI) network information, predict the hub genes in pathology of periodontitis.

**Results:**

In total 668 DMGs were sorted and identified from the data set, which included 621 hypo-methylated genes and 47 hyper-methylated genes. Through the function and ontology analysis, these 668 genes are mainly classified into intracellular signaling pathway, cell components, cell–cell interaction, and cellular behaviors. The pathway analysis showed that the hypo-methylated genes were mostly enriched in the pathway of cGMP–PKG signaling pathway; RAF/MAP kinase; PI3K–Akt signaling pathway, while hyper-methylated genes were mostly enriched in the pathway of bacterial invasion of epithelial cells; sphingolipid signaling pathway and DCC mediated attractive signaling. The PPI network contained 630 nodes and 1790 interactions. Moreover, further analysis identified top 10 hub genes (APP; PAX6; LPAR1; WNT3A; BMP2; PI3KR2; GATA4; PLCB1; GATA6; CXCL12) as central nodes that are involved in the immune system and the inflammatory response.

**Conclusions:**

This study provides comprehensive information of methylation status of genes to the revelation of periodontitis pathogenesis that may contribute to future research on periodontitis.

**Supplementary Information:**

The online version contains supplementary material available at 10.1186/s40729-022-00420-8.

## Introduction

Periodontitis is a chronic inflammatory disease that compromises the integrity of the tooth-supporting tissues, including gingiva, periodontal ligament and alveolar bone. Research have revealed that periodontitis could occur in the 20% to 50% of people around the world [1]. Moreover, the presence or history of periodontitis is determined to be a potential risk factor for various complications in dental implant therapy, including peri-implantitis, marginal bone loss, and then jeopardizes the longevity of dental implants. In recent years, more and more research have demonstrated the association between periodontitis and peri-implantitis. [2, 3]. Karoussis et al. suggested that the incidence of peri-implantitis was 4–5 times higher in individuals with a history of chronic periodontitis, than in individuals with no history of periodontitis [4]. Thus, prevention and therapy on periodontitis could contribute health of teeth and avoid the loss of dental implants.

Periodontitis usually is caused by gingivitis, which is induced by untreated abundant plaque and calculus beneath the gingival margin. The pathology of periodontitis is characterized by infiltration of inflammatory cells, and untreated periodontitis could result in the bone loss, destruction of ligaments the health teeth [5]. Moreover, some studies have found the periodontitis could increase risk of cancer [6], diabetes [7], cardiovascular diseases [8].

Some risk factors related to periodontitis have been identified, including smoking, diabetes [9], alcohol consumption, lack of maintenance [10, 11]. However, the pathophysiology of periodontitis remains controversial. In addition, there is still no ideal biomarker to predict the progression of periodontitis, and lack of efficient therapy for periodontitis with gingival recession. Although the presence of micro-organisms has been identified in the periodontitis, such as Aggregatibacter actinomycetemcomitans, Porphyromonas gingivalis, Eikenella corrodens, the true ‘pathogens’ in periodontitis has been elusive. Many researchers believed that immune response and the inflammatory processes induced by micro-organisms play more important roles in pathology of periodontitis.

In recent years, the progress in the genetic data technology make it possible that the researchers can explore the pathogenesis of periodontitis using genetic method, which may can help us identify the critical genes or pathways in periodontitis, then these alternated genes can be used as a diagnosis biomarkers or treatment targets. Besides the genetic changes, the epigenetic changes could play an important role in the pathology of periodontitis. In fact, it has known that epigenetic factors, such as methylation, acetylation participate the pathological process of inflammation response [12]. For example, decreased methylation of histone H3K27 triggers expression of specific inflammatory genes [13]. Therefore, we speculated that methylation is involved in the pathology of periodontitis. In addition, investigation on the gene methylation in pathology of periodontitis can provide more genetic information to understand pathology of periodontitis thoroughly, which then might contribute to develop the efficient therapy against periodontitis. In fact, multiple studies have investigated global DNA methylation patterns in periodontitis, and they believed that multiple methylation alterations were responsible for periodontitis [14–17]. However, no further research deeply analyzed and elucidated methylation mechanisms in periodontitis. Therefore, in the present study, via an integrated bioinformatic analysis, our objective is to re-analyze methylation status of genes from the data sets of periodontitis, to determine differentially methylated genes (DMGs) and significant pathways in periodontitis. Identifying DMGs and enriching their biological functions/key pathways will provide more accurate information regarding the pathogenesis of periodontitis, which will contribute to further research and therapies.

## Materials and methods

### Data set and DMG identification

To obtain the methylation data of periodontitis tissues in Gene Expression Omnibus (GEO) database, our inclusion criteria: (1) human periodontitis disease, (2) gingival tissues, and (3) methylation analysis data. We searched the in GEO, using the key words “periodontitis”, “disease”, “tissue”, “Homo sapiens” and “methylation”. In addition, we located two data sets GSE173081 and GSE58493. However, the samples of data set GSE58393 were collected from oral swab, instead of gingival tissues, thus we excluded the data set GSE58393. Therefore, the data set GSE173081 were included in our study for further analysis. The microarray data for GSE173081 were based on the GPL21145 Platform (Infinium MethylationEPIC) and included gingival tissues from 12 periodontitis patients, and 12 healthy subjects. The information of GPL21145 Platform provided by Illumina Inc was used to convert spot ID into official gene symbol. The periodontitis samples were collected from the lesions of periodontitis, while the controls were the normal gum tissues collected from the healthy subjects. Both the lesions of periodontitis and normal gum tissues were confirmed by pathologist. The raw data of methylation profiling by genome tiling array were download and analyzed in GEO2R. Classical t test and level of fold changes were used to identify the DMGs, as the following criteria: *p* < 0.05 and [logFC] > 1. Thereafter, the identified DMGs were saved for further analysis.

### Enrichment of gene ontology and pathway analysis

After identified, the DMGs were uploaded in the multiple online databases to perform the bioinformatics analysis, including KEGG, REACTOME, BIOCYC, and DAVID, with the cut off value: *p* < 0.05. DAVID (The Database for Annotation, Visualization, and Integrated Discovery, V6.8) is an online database provide the biological meaning through integrated analysis of large gene set on gene ontology, genome function, and genome related diseases. In present study, we uploaded the identified DMGs into DAVID, and then to explore the potential biological themes, particularly GO terms, functional-related groups.

The data base of KEGG, REACTOME, BIOCYC are the collection of databases dealing with biological pathways, genomes, drugs, diseases. All these three databases were used to explore the related biological pathways in periodontitis. The identified DMGs were employed into these 3 databases, respectively, and the significant pathway were identified with cutoff value *p* < 0.05. Then the identified the potential pathways from each database were compared and combined.

### Integration of PPI network generated based on DMGs

To investigate the interaction of gene network in periodontitis, the identified DMGs were uploaded into online database of Retrieval of Interacting Genes (STRING; http://string-db.org) [18], and then the PPI network information between DMGs were generated and downloaded (PPI score > 0.4). Thereafter the data of PPI were reloaded into Cytoscape for further analysis, which is a bioinformatics platform for constructing and visualizing molecular interaction networks.

To identify the hub gene of PPI network, a software “cytoHubba” was installed in the Cytoscape and then employed, which can screen hub genes of PPI network through Betweenness, BottleNeck, Closeness, Clustering Coefficient, Degree, DMNC, EcCentricity, EPC, MCC, MNC, Radiality and Stress based on shortest paths [19].

## Results

### Identification of DMGs in periodontitis

**Fig. 1:**
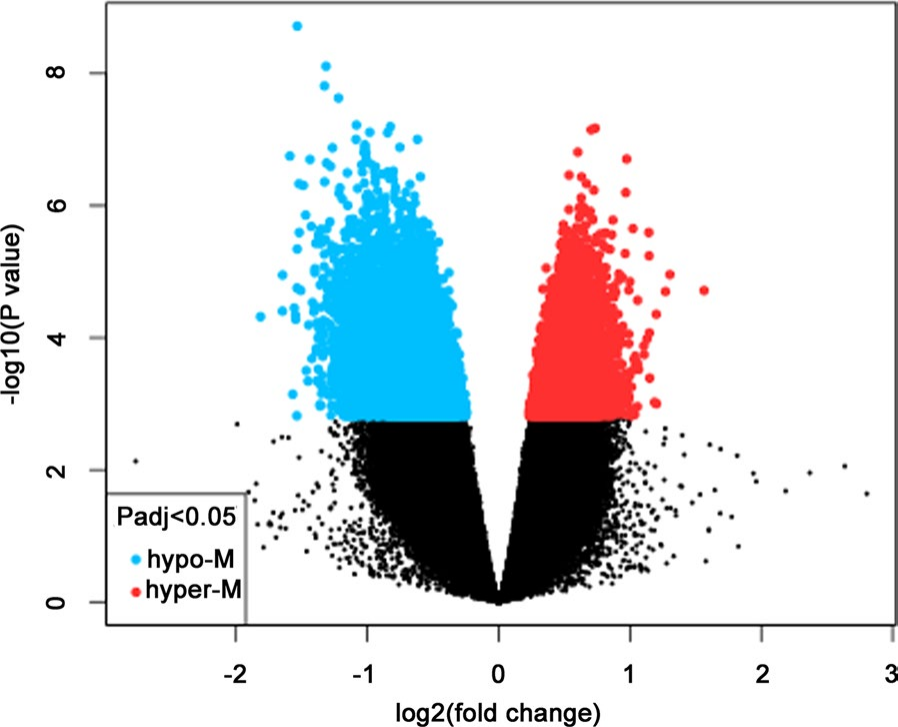
Volcano plot of methylation difference in periodontitis. A total of 621 hypo-methylated and 47 hyper-methylated genes in periodontitis with *p* < 0.05 and [logFC] > 1. Hyper-methylated genes were represented by red point in the right side, while hypo-methylated genes were represented by blue point in the left side

After standardization of microarray data, the 668 DMGs were identified in total, including 47 hypermethylation genes and 621 hypomethylation genes between periodontitis samples and healthy samples, for further analysis (Fig. 1, Table 1, and Additional file 1).

**Table 1:** Identified DMGs from data sets in periodontitis tissues

DMGs	Gene list
Hyper-methylated	AATK, ABLIM2, ADARB2, AHR, ARHGAP10, ARHGAP39, ARPC1B,CCDC125, CCZ1B, CDC42BPA, CKLF-CMTM1, CMTM7, CSGALNACT2, DCTN5, DEGS2, DGKQ, DOCK1, ELFN1-AS1, ELFN2, FARS2, GPR155, GSTT1, HCG14, KCNJ6, KIAA1462, KNDC1, LEMD3, LRCH4, MCM5, MYO18A, NXT1, OCA2, OR8B8, OS9, PAPD7, PCM1, PLD6, PRKCZ, RET, RNF39, SOX2-OT, SPTLC2, ST5, TLE2, TMEM9B, TRPS1, TTC19
Hypo-methylated	RBM20, RBM24, RBP7, RCAN1, RELN, RFX6, RHCG, RHOU, RIMS4, RNF150, RNF217, ROBO2, ROR1, RORA, SALL1, SATB2, SCARF2, SCGB3A1, SCN4B, SDK2, SEC23IP, SEMA3C, SEMA5A, SEMA6D, SH2D4A, SH3BP4, SH3GL3, SHC2, SHISA8, SIM1, SIM2, SIX3-AS1, SLAIN1, SLC15A2, SLC1A6, SLC22A17, SLC24A4, SLC25A25, SLC30A2, SLC32A1, SLC35D2, SLC38A3, SLC40A1, SLC6A2, SLC6A3, SLC9A2, SLIT1, SLIT3, SLITRK5, SLMAP, SMTNL2, SNCA, SND1, SNTG2, SNX32, SOWAHC, SOX14, SOX17, SPEG, SPOCK1, SPRY2, SPTB, SRF, SRP68, SRRM4, SSH1, ST6GAL2, ST8SIA2, STBD1, STC2, STK17B, STK24, STK32B, STK32C, SULF2, SYNDIG1L, SYNGR1, SYNM, SYPL2, SYT6, SYT9, TACC2, TACR1, TAF3, TBCD, TBR1, TBX18, TBX2, TBX2-AS1, TFAP2A-AS1, TFAP2C, TH2LCRR, THADA, THBS4, TJP1, TLL1, TLX2, TMEM132E, TMEM150B, TMEM178A, TNFRSF1B, TNIP3, TPBGL, TRAF3, TRAM2-AS1, TRIL, TRIO, TRPC6, TSPAN33, TTC34, TUB, UBE4B, UBL3, UCP1, UGGT2, UNC5A, USH1C, USP16, USP2, USP44, USP6NL, VAC14, VENTX, VIPR2, VPS53, VWC2, WBP1L, WBSCR17, WNT3A, WNT7B, WNT9B, WT1-AS, YIPF4, ZBTB8B, ZC3H3, ZFP28, ZFPM2, ZFR2, ZIC2, ZIK1, ZNF285, ZNF354C, ZNF503-AS2, ZNF542P, ZNF577, ZNF655, ZNF662, ZNF667, ZNF677, ZNF697, ZNF718, ZNF781

### DMG GO analysis

**Fig. 2:**
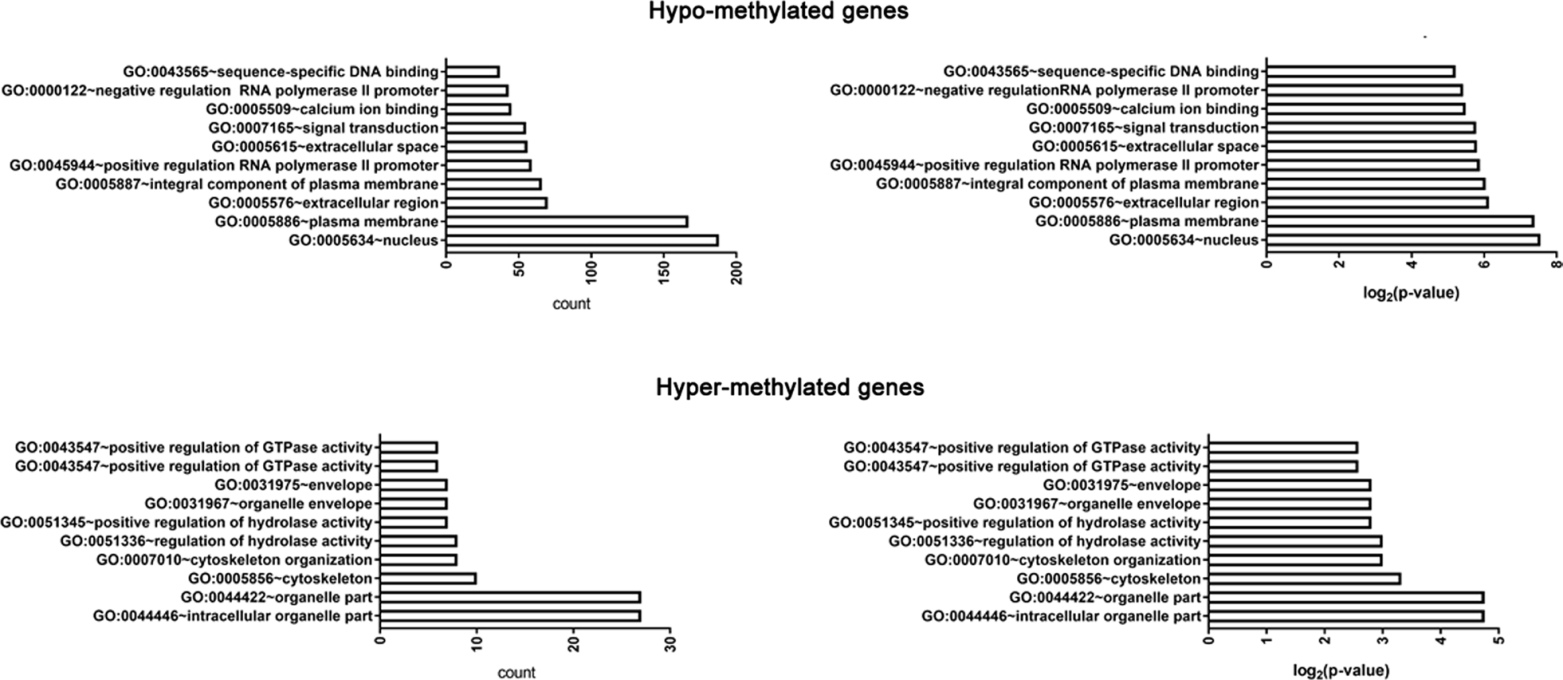
Top 10 Gene Ontology analysis and significantly enriched GO terms of the candidate DMGs. Up, GO analysis classified the common hypo-methylated DMGs; down, GO analysis classified the common hyper-methylated DMGs,

After candidate DMGs were identified, the gene list was uploaded into DAVID online database to perform gene ontology analysis. The results showed that the DMGs were enriched mainly into following functional groups: intracellular signaling pathway, cell components, cell–cell interaction, and cellular behaviors, etc. (Fig. 2, Tables 2, 3, and Additional file 2). After the further analysis, intracellular signaling pathway group were mainly classified into subgroups of signal transduction, transcription from RNA polymerase II promoter, regulation of transcription, intracellular signal transduction, MAPK cascade, Wnt signaling pathway, phosphatidylinositol 3-kinase signaling, GTPase activity, protein kinase C signaling. In the cell components group, all the related genes were classified in the subgroups of integral component of plasma membrane, actin binding, actin cytoskeleton, organelle part. In addition, the cell–cell interaction group were mainly classified into subgroups of proteinaceous extracellular matrix, single organismal cell–cell adhesion, proteinaceous extracellular matrix, cell adhesion, cell junction. The cellular behavior group were mainly classified into subgroups of cell proliferation: regulation of cell proliferation, migration, endocytosis.

**Table 2:** Top 10 significantly enrichment analysis of hyper-methylated DMGs in periodontitis tissues

Term	Description	Count	Percentage	*p* value
GO:0044446	Intracellular organelle part	27	55.1020408	0.02155529
GO:0044422	Organelle part	27	55.1020408	0.02927057
GO:0005856	Cytoskeleton	10	20.4081633	0.04142009
GO:0007010	Cytoskeleton organization	8	16.3265306	0.01722379
GO:0051336	Regulation of hydrolase activity	8	16.3265306	0.03663579
GO:0051345	Positive regulation of hydrolase activity	7	14.2857143	0.02043463
GO:0031967	Organelle envelope	7	14.2857143	0.04659827
GO:0031975	Envelope	7	14.2857143	0.04742665
GO:0043547	Positive regulation of GTPase activity	6	12.244898	0.01054484
GO:0043547	Positive regulation of GTPase activity	6	12.244898	0.01668003

**Table 3:** Top 10 significantly enrichment analysis of hypo-methylated DMGs in periodontitis tissues

Term	Description	Count	Percentage	*p* value
GO:0005634	Nucleus	188	29.8412698	0.02328831
GO:0005886	Plasma membrane	167	26.5079365	3.51E-05
GO:0005576	Extracellular region	70	11.1111111	0.00245553
GO:0005887	Integral component of plasma membrane	66	10.4761905	5.91E-04
GO:0045944	Positive regulation of transcription from RNA Polymerase II promoter	59	9.36507937	2.03E-06
GO:0005615	Extracellular space	56	8.88888889	0.01627481
GO:0007165	Signal transduction	55	8.73015873	0.00212399
GO:0005509	Calcium ion binding	45	7.14285714	1.42E-05
GO:0000122	Negative regulation of transcription from RNA polymerase II promoter	43	6.82539683	7.65E-05
GO:0043565	Sequence-specific DNA binding	37	5.87301587	6.08E-06

### Signaling pathway enrichment analysis

**Fig. 3:**
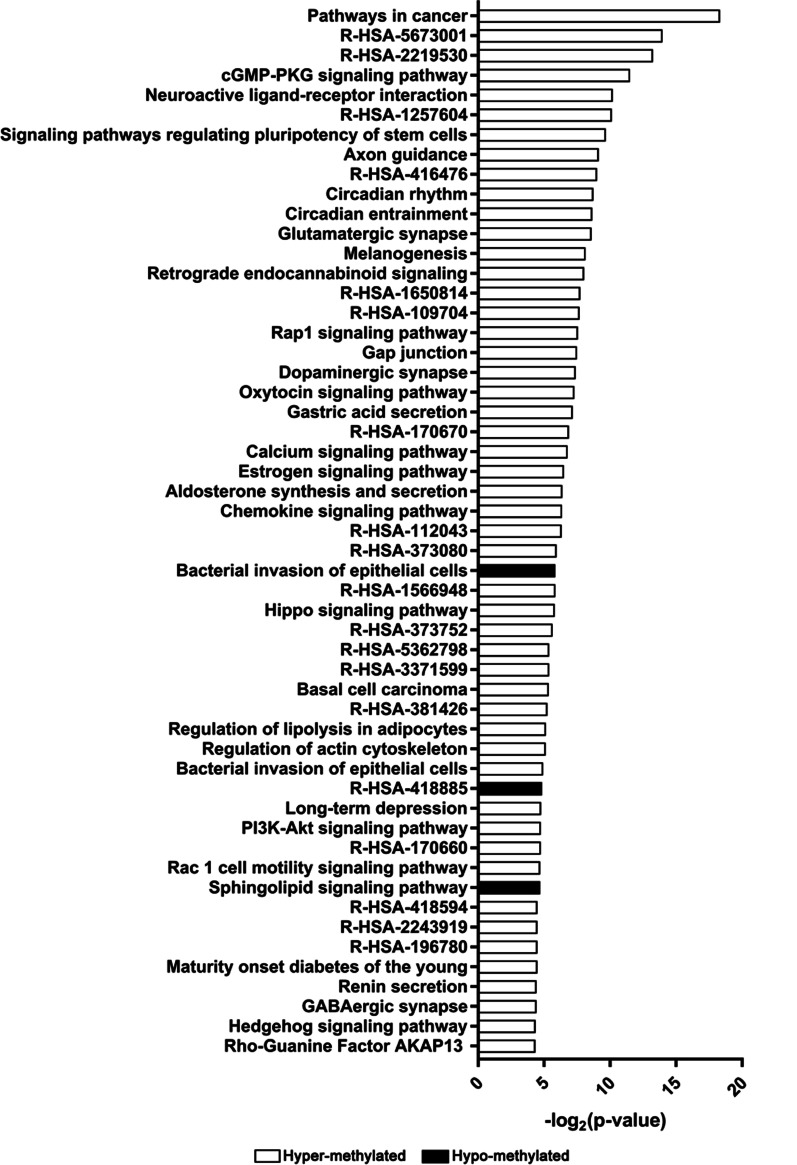
Significantly enriched pathway terms of the candidate genes in periodontitis. Functional and signaling pathway enrichment analysis were conducted using the KEGG pathway, Reactome, and BioCyc online websites

To analyze the involved functional and signaling pathways of DMGs, multiple online databases including KEGG, REACTOME, and BIOCYC, were employed. In addition, the results showed that the hypo-methylation genes were mainly enriched in the pathways of: cancer pathway; cGMP–PKG signaling pathway; RAF/MAP kinase; PI3K–Akt signaling pathway; neuroactive ligand–receptor interaction; stem cells pluripotency, etc., while hyper-methylation genes were mainly enriched in the pathways of: bacterial invasion of epithelial cells; sphingolipid signaling pathway and DCC mediated attractive signaling (Fig. 3, Table 4, and Additional file 3).

**Table 4:** Signaling pathway enrichment analysis of DMGs in periodontitis tissues

Pathway identifier	Term	Regulation	Count	*p* value
hsa05100	Bacterial invasion of epithelial cells	Hyper-methylated	3	0.017267317
hsa04071	Sphingolipid signaling pathway	Hyper-methylated	3	0.038486205
R-HSA-418885	DCC mediated attractive signaling	Hyper-methylated	2	0.034928066
hsa05200	Pathways in cancer	Hypo-methylated	31	3.04E-06
hsa04080	Neuroactive ligand–receptor interaction	Hypo-methylated	20	8.45E-04
hsa04151	PI3K–Akt signaling pathway	Hypo-methylated	18	0.0369
hsa04022	cGMP–PKG signaling pathway	Hypo-methylated	15	3.04E-06
hsa04015	Rap1 signaling pathway	Hypo-methylated	15	0.0053
R-HSA-5673001	RAF/MAP kinase cascad	Hypo-methylated	14	6.21E-05
R-HSA-416476	G alpha (q) signalling	Hypo-methylated	14	0.001925
R-HSA-418594	R-HSA-418594	Hypo-methylated	14	0.044195
hsa04550	Signaling pathways regulating pluripotency of stem cells	Hypo-methylated	13	0.0012
hsa04020	Calcium signaling pathway	Hypo-methylated	13	0.0091
hsa04062	Chemokine signaling pathway	Hypo-methylated	13	0.0121
hsa04810	Regulation of actin cytoskeleton	Hypo-methylated	13	0.0287
hsa04360	Axon guidance	Hypo-methylated	12	0.0018
hsa04921	Oxytocin signaling pathway	Hypo-methylated	12	0.0064
hsa04724	Glutamatergic synapse	Hypo-methylated	11	0.0026
hsa04728	Dopaminergic synapse	Hypo-methylated	11	0.006
hsa04390	Hippo signaling pathway	Hypo-methylated	11	0.0179
R-HSA-2219530	Constitutive Signaling by Aberrant PI3K	Hypo-methylated	10	1.03E-04
R-HSA-1257604	PIP3 activates AKT signaling	Hypo-methylated	10	8.96E-04
hsa04713	Circadian entrainment	Hypo-methylated	10	0.0025
hsa04916	Melanogenesis	Hypo-methylated	10	0.0035
hsa04723	Retrograde endocannabinoid signaling	Hypo-methylated	10	0.0038
hsa04540	Gap junction	Hypo-methylated	9	0.0055
hsa04915	Estrogen signaling pathway	Hypo-methylated	9	0.0111
R-HSA-1650814	Collagen biosynthesis and modifying enzymes	Hypo-methylated	8	0.004679
hsa04971	Gastric acid secretion	Hypo-methylated	8	0.007
hsa04925	Aldosterone synthesis and secretion	Hypo-methylated	8	0.0121
hsa05100	Bacterial invasion of epithelial cells	Hypo-methylated	7	0.0326
hsa04727	GABAergic synapse	Hypo-methylated	7	0.0466
R-HSA-109704	PI3K Cascade	Hypo-methylated	6	0.004858
R-HSA-373080	Class B/2 (Secretin family receptors)	Hypo-methylated	6	0.016312
hsa04710	Circadian rhythm	Hypo-methylated	6	0.0023
hsa05217	Basal cell carcinoma	Hypo-methylated	6	0.0247
hsa04923	Regulation of lipolysis in adipocytes	Hypo-methylated	6	0.0284
hsa04730	Long-term depression	Hypo-methylated	6	0.0368
hsa04924	Renin secretion	Hypo-methylated	6	0.0466
R-HSA-170670	Adenylate cyclase inhibitory pathway	Hypo-methylated	4	0.008461
R-HSA-112043	PLC beta mediated events	Hypo-methylated	4	0.012432
R-HSA-1566948	Elastic fibre formation	Hypo-methylated	4	0.017303
R-HSA-373752	Netrin-1 signaling	Hypo-methylated	4	0.020083
R-HSA-381426	Regulation of Insulin-like Growth Factor	Hypo-methylated	4	0.026335
hsa04950	Maturity onset diabetes of the young	Hypo-methylated	4	0.0446
hsa04340	Hedgehog signaling pathway	Hypo-methylated	4	0.049
h_rac1Pathway	Rac 1 cell motility signaling pathway	Hypo-methylated	4	0.038391
R-HSA-5362798	Release of Hh-Np from the secreting cell	Hypo-methylated	3	0.023963
R-HSA-3371599	Defective HLCS causes multiple carboxylase deficiency	Hypo-methylated	3	0.023963
R-HSA-170660	Adenylate cyclase activating pathway	Hypo-methylated	3	0.036964
R-HSA-196780	Biotin transport and metabolism	Hypo-methylated	3	0.044264
R-HSA-2243919	Crosslinking of collagen fibrils	Hypo-methylated	3	0.044264
h_akap13Pathway	Rho-Selective Guanine Exchange Factor AKAP13 Mediates Stress Fiber Formation	Hypo-methylated	3	0.049203

### PPI network construction and module analysis

**Fig. 4:**
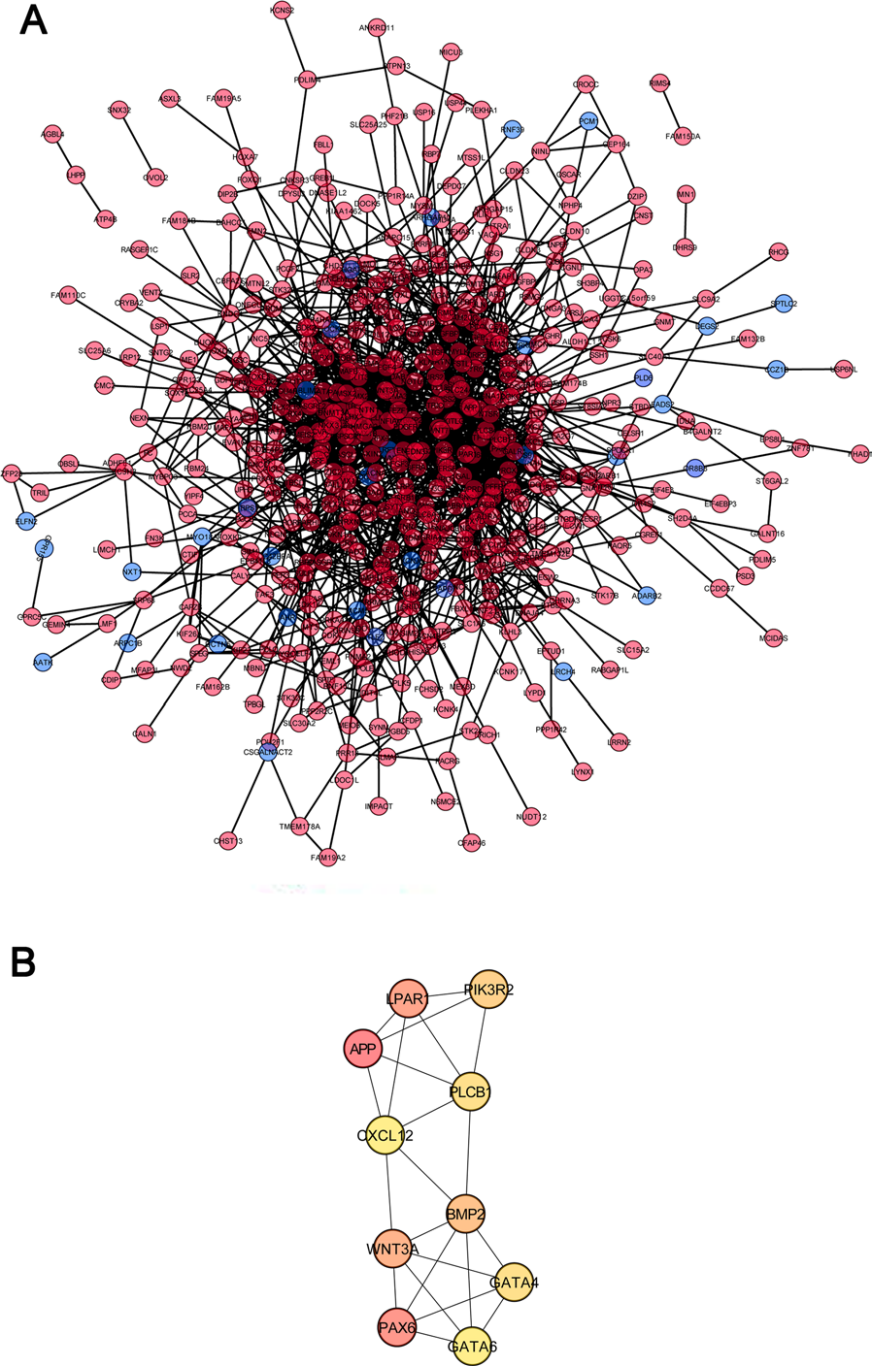
Protein–protein interaction (PPI) network and modular analysis of the DMGs in periodontitis. **A** Using the STRING online database, a total of 630 nodes (red represents hypo-methylated genes, and blue represents hyper-methylated genes) and 1790 interactions were identified in the DMG PPI network. **B** Top 10 genes in network string interactions by Degree method

To further investigate the interaction of DMGs in pathology of periodontitis, the PPI network was generated based on the gene interaction database of string. In total, 630 nodes and 1790 interactions were screened to establish the PPI network (Fig. 4).

Further analysis revealed the most significant module, and the top 10 genes (APP; PAX6; LPAR1; WNT3A; BMP2; PI3KR2; GATA4; PLCB1; GATA6; CXCL12 in Hypo-HGs) were identified as hub genes (Table 5).

**Table 5:** Top 10 genes in network string interactions ranked by degree method

Rank	Gene name	Score
1	APP	61
2	PAX6	43
3	LPAR1	33
4	WNT3A	31
5	BMP2	30
6	PIK3R2	29
7	GATA4	27
7	PLCB1	27
9	GATA6	26
9	CXCL12	26

## Discussion

In our integration analysis, multiple gene pathways are involved in the pathology of periodontitis, including Wnt pathway, PI3K–Akt signaling pathway, bacterial invasion of epithelial cells and so on. Therefore, the we suggest that inflammation response related to the intracellular signaling pathway plays a key role in the periodontitis pathology.

In the pathology of periodontitis, recent research has revealed the existence of poly micro-organisms in the subgingival region, and then form the biofilm. Both bacteria and biofilm can directly recruit the immune cells, including NK cells, neutrophil cells, and lymphocyte cells, which then release various cytokines responding to the infection of bacteria [20, 21]. The inflammatory response could trigger the pathological changes in the subgingival region, and then the periodontitis induces the ecological successions in the subgingival area, destroys structures that hold the tooth, and results in tooth loss. For the patients who received dental implant, periodontitis might induce the postoperative infections and marginal bone loss, both of which resulted in implant failure [22]. Especially patients with periodontitis and with ≥ 4 implants, exhibited higher odds ratios (ORs) for moderate/severe peri-implantitis [23]. In addition, in one study by Dreyer et al., the data showed that the median prevalence of peri-implantitis was 14.3% for patients with a history of periodontitis [24]. Therefore, exploring the pathology of periodontitis will contribute to develop treatment strategy, and then benefit the health of dental implant.

During the inflammatory response of periodontitis, the lymphocytes, macrophage and neutrophils are regulated by expression of multiple genes, such as ARHGAP10, ARPC1B, DOCK1, FGF2/4, TNFRSF1B, NXT1, and AHR, all of which were identified in our analysis. In the KEGG analysis on the genes with hyper-methylation, the pathway related to bacterial invasion of epithelial cells was identified, including genes of ARHGAP10, ARPC1B, DOCK1. In human, ARPC1B encode protein of Actin-related protein 2/3 complex subunit 1B, which normally is one of components of actin filaments in cells, and contributes to maintain the normal immune function of cells. Loss the expression ARPC1B could serve in inflammatory disease. Kahr et al. reported that loss the expression of ARPC1B resulted in the abnormality of platelet, and then contributed to inflammatory disease in human and mice model [25], while Kuijpers et al. found that ARPCIB deficiency can cause immunodeficiency and other inflammation disease [26]. ARHGAP10 is the gene that encodes Rho GTPase-activating protein 10. A study has shown that the expression of ARHGAP10 could be related to the infection of Listeria, in which knockdown the expression of ARHGAP10 impaired alpha-catenin recruitment, and then inhibited the invasion of Listeria [27]. DOCK1 is the gene encoding dedicator of cytokinesis, and a study has shown that expression loss of DOCK1 could lead to immunodeficiency [28]. In sum, all these 3 genes were related to the maintain normal immune function, and dysregulation of these 3 genes could affect the immune defense, which then results in the vulnerable to the infection. In addition, using the gene ontology, our data have shown that most hyper-methylated genes are related intracellular organelle part, organelle part, such as OCA2, DCTN5, NXT1, ARPC1B, AHR, etc. we speculated the hyper-methylation of these genes could attribute to the cell injury by infection, inflammation, oxidase burst etc. The cellular stress by infection lead to cell injury, and then resulted in the organelle related gene down-regulated [29, 30].

In our data, most of identified hypo-methylated genes were related to the inflammation, cell mobility, classic cell signaling, cell proliferation, cell junction, all of which regulate various bio-behavior. For example, we found the hypo-methylated level of FGF2 and 4, both of which have been identified in other studies on both periodontitis and peri-implantitis. In research on inflammation, the expression of FGF2 can regulate the angiogenesis [31], while FGF4 could promote the vascular permeability, angiogenesis in the inflammation response [32]. Moreover, Coelho et al. suggested that increased FGF 2 and 4 increased the risk of peri-implantitis [33]. Both FGF2 and 4 may be potential therapeutic strategies for the treatment of periodontitis [34–37]. D’Mello et al. found that FGF2 can promote proliferation of bone marrow stromal cells [37]. Yoshida et al. found that treatment with recombinant human FGF2 and deproteinized bovine bone mineral clearly improved probing pocket depth and clinical attachment in the patients with chronic periodontitis [34]. In addition, Son et al. suggested that FGF-4 can promote cellular viability and osteogenic differentiation of stem cell spheroids [36].

The further PPI analysis has revealed some of genes play a key role in pathogenesis in periodontitis, including WNT3A, BMP2, CXCL12 and PI3KR2. More interestingly, some of these key genes also play a role in the pathology of peri-implantitis, such as WNT3A, PI3K, BMP2, which indicated the interaction between periodontitis and peri-implantitis [38]. WNT3A, as one of Wnt family members, participates in various bio-behaviors of cells, including inflammation. Although polymicro-organism existence in periodontitis has been demonstrated, Porphyromonas gingivalis is one of bacteria most investigated in periodontitis. Both gingival epithelial cells and macrophage internalize the Porphyromonas gingivalis in to the cells, and then multiple pathways of cells could be activated to maintain the survival of host cells, including Wnt, PI3K/Akt. Moreover, the activation of Wnt and PI3K/Akt pathways could induce the production of inflammatory cytokines, such as IL-1, IL-6 and TNF-α1 [39]. In fact, previous studies have revealed that Wnt3A played a role in the oral inflammation pathology. For example, Jang et al. suggested that Lipopolysaccharid induced the inflammatory response through activation of Wnt3A in human bronchial epithelial cells [40]. In addition, Tebroke et al. suggested that activated Wnt3A can induce inflammatory cytokines release in human mast cells, such as IL-8 [41]. Lü et al. suggested that activation of Wnt3A promoted infiltration of inflammatory cells, and production of pro-inflammatory cytokines in oral cavity [42]. In addition, the dysregulation of Wnt pathway could contribute to the pathology of peri-implantitis. Instead of Wnt3A, Wnt5A expression was found to be upregulated in the gingival tissues with peri-implantitis [43]. However, the effect of Wnt3A could be controversial. In a study on periodontitis of rat model, the researchers found that Wnt3A activity was decreased [44]. We speculate the bias could be due to the origin of samples. In the periodontitis, the activated Wnt3A contributed to the inflammatory response. However, Wnt3A can promote the bone formation around the implants. Thus, in periodontitis pathology, the decreased expression of Wnt3A could affect the repair of bone by restriction on bone formation.

The PI3KR2 gene encodes the p85β which is a regulatory subunit of class PI3K for the activation, while Phosphatidylinositol-3-kinase (PI3K)/AKT/mammalian target of rapamycin (mTOR) signaling is one of the most important intracellular pathways, which regulates cell proliferation, motility, differentiation, etc. Previous studies have shown that PI3KR2 contributes to the inflammatory pathology via multiple ways. Balakrishnan et al. found that IL-6 can regulate the DNA methylation status of PI3KR2 of cells [45], while IL-6 is one of the most important players in the pathology of inflammation. Eräsalo et al. suggested that PI3K Inhibitors LY294002 reduced inflammation response and activation of macrophages through blocking multiple subunits of PI3K, including PI3KR2 [46]. In addition, in our study, the analysis showed the status of PI3KR2 could play a key role in the pathology of periodontitis, while Abdelgawad et al. found that up-regulated expression of gene PI3K was related increased cell viability of human periodontal ligament stem Cells [47]. In fact, PI3K also plays a role in the pathology of peri-implantitis. Zhu et al. suggested that magnesium-modified titanium could directly induce the activation of PI3K [48]. In addition, in another study on peri-implantitis, Zhang et al. found the PI3K–Akt pathway were significantly upregulated using bioinformatics methods [49]. Thus more research is needed to explore the pathophysiology mechanism of PI3KR2 in periodontitis, and its related signaling pathways. We suggested that activation of both Wnt3A and PI3KR2 could promote the inflammatory response in tissues, which then lead to the pathological changes and loss of teeth eventually. In addition, Porphyromonas gingivalis can also regulate the expression of adhesion protein-like BMP2, which was identified in present study, to inhibit the adhesion and transmigration of leukocytes. Kang et al. suggested that Porphyromonas gingivalis degraded BMP2 in epithelial cell of gingival, and then disrupted interaction between neutrophils and epithelial cell [50].

In addition, our results showed the genes of TNFRSF1B and NPR3 were hypo-methylated in the periodontitis. TNFRSF1B is the gene encoding the receptor of TNF, which is a paracrine and endocrine cytokine, produced by activated macrophages, monocytes, and other types of immune cells. TNF can bind the receptor, including TNFRSF1B, on target cell surface and exert the various function, including cytotoxicity, intracellular pathogens defense, and immune cell activation. Especially during the infection of virus, bacteria or parasites, TNF-α can activate CD4 T cells, enhance the kill capacity of macrophage, induce death of infected cells [51–53]. NPR3 is a peptide related to function of vessels. The expression of NPR3 can participate into inflammation response. Bruckmeier et al. found that the expression of NPR3 could be related to the activity of human peripheral blood monocytic cells [54]. Thus, the hypo-methylated status of NPR3 could promote its expression, which then participate in the inflammation process. However, a study by Zhou et al. investigated the dysregulation of genes in periodontitis, and they found that NRP3 expression had significant between periodontitis and peri-implantitis [55], which indicated the potential difference in pathology and gene expression prolife between periodontitis and peri-implantitis, and need more research to explore.

## Conclusions

Taken together, our findings provide epigenetic information about the pathology of periodontitis, which could be diagnostic biomarkers or therapy targets.

## Supplementary Information

**Additional file 1.** List of gene name abbreviations.

**Additional file 2.** Gene ontology results.

**Additional file 3.** Signaling pathway enrichment analysis.

## Data Availability

Not applicable.
